# Interactions of Galloylated Polyphenols with a Simple Gram-Negative Bacterial Membrane Lipid Model

**DOI:** 10.3390/membranes14020047

**Published:** 2024-02-08

**Authors:** Ryan T. Coones, Maarit Karonen, Rebecca J. Green, Richard Frazier

**Affiliations:** 1 School of Chemistry, Food and Pharmacy, University of Reading, Harry Nursten Building, Pepper Lane, Whiteknights, Reading RG6 6DZ, UK; ryan.coones@npl.co.uk (R.T.C.); r.a.frazier@reading.ac.uk (R.F.); 2 Natural Chemistry Research Group, Department of Chemistry, University of Turku, 20014 Turku, Finland; maarit.karonen@utu.fi

**Keywords:** polyphenol, tannin, phospholipid, vesicle, differential scanning calorimetry

## Abstract

Differential scanning calorimetry (DSC) was used to explore the interactions of isolated polyphenolic compounds, including (-)-epigallocatechin gallate ((-)-EGCg), tellimagrandins I and II (Tel-I and Tel-II), and 1,2,3,4,6-penta-*O*-galloyl-d-glucose (PGG), with a model Gram-negative bacterial membrane with a view to investigating their antimicrobial properties. The model membranes comprised 1,2-dipalmitoyl-sn-glycero-3-phosphoethanolamine (DPPE) and 1,2-dipalmitoyl-sn-glycero-3-phospho-(1’-rac-glycerol) (DPPG), fabricated to mimic the domain formation observed in natural membranes, as well as ideally mixed lipid vesicles for the interaction with (-)-EGCg. Polyphenols induced changes in lipid mixing/de-mixing depending on the method of vesicle preparation, as was clearly evidenced by alterations in the lipid transition temperatures. There was a distinct affinity of the polyphenols for the DPPG lipid component, which was attributed to the electrostatic interactions between the polyphenolic galloyl moieties and the lipid headgroups. These interactions were found to operate through either the stabilization of the lipid headgroups by the polyphenols or the insertion of the polyphenols into the membrane itself. Structural attributes of the polyphenols, including the number of galloyl groups, the hydrophobicity quantified by partition coefficients (logP), and structural flexibility, exhibited a correlation with the temperature transitions observed in the DSC measurements. This study furthers our understanding of the intricate interplay between the structural features of polyphenolic compounds and their interactions with model bacterial membrane vesicles towards the exploitation of polyphenols as antimicrobials.

## 1. Introduction

Polyphenols are plant specialized metabolites, which include the classes of flavonoids, stilbenes, lignans, and tannins, that have been identified as compounds with biological significance in humans and animals and which are highly structure-dependent [1,2]. In the context of animal nutrition, dietary polyphenols have been shown to help solubilize proteins in animal feed, which aids protein absorption in livestock and helps prevent and treat parasitic nematodes (as anthelmintics) and gastrointestinal symptoms [3]. They are reported to possess antimicrobial effects against several pathogens and could, therefore, be of interest as an alternative to antibiotics [4,5]. With respect to both antimicrobial and anthelmintic activities, it is relevant that there are also reports of polyphenols that have interactions with biologically significant lipid membranes [6,7,8,9,10,11] via hydrogen bond formation, with the hydroxyl moieties serving as the hydrogen bond donors and the phospholipid bound oxygen atoms as the acceptors [12]. Thus, polyphenols could exhibit their antimicrobial properties via targeting lipid membranes, for example, to disrupt membrane permeability [13].

While polyphenols are well-studied regarding their interactions with proteins and protein-binding capacities, less is known about their interactions with other biomacromolecules, including lipids [14]. The interactions of polyphenols with lipid and cell membranes are important, as these partially determine their biological activity [6]. Previous studies, based mainly on lipid bilayers as membrane models, have shown that the interaction between polyphenols and lipid bilayers is affected by the hydrophobicity of the polyphenols and that the intermolecular interaction forces stabilizing the location and orientation of the polyphenol in the lipid bilayer are important [13,15]. The interactions have been found to be dependent both on the structures of the polyphenols and the compositions of the membrane lipids involved. Therefore, more detailed studies on polyphenol–lipid interactions using different model polyphenols, different model lipids, and different techniques are needed to understand the complex processes, the intensity of the interactions, and the forces included [13,16].

**Figure 1:**
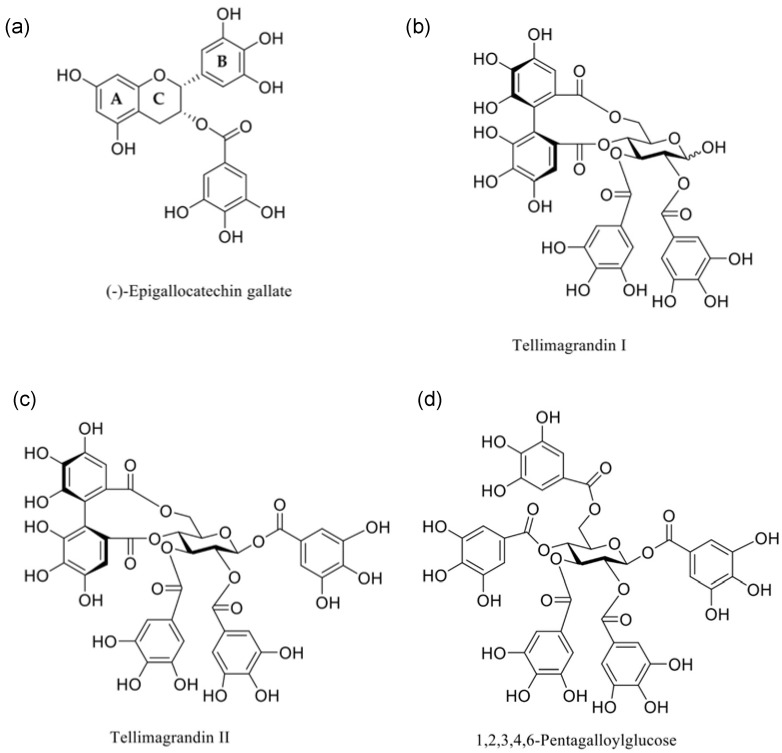
Chemical structures of the polyphenols studied in this paper: (**a**) (–)-epigallocatechin-3-gallate ((-)-EGCg), (**b**) tellimagrandin I, (**c**) tellimagrandin II (Tel-I and Tel-II, respectively), and (**d**) 1,2,3,4,6-pentagalloylglucose (PGG).

The tendency for polyphenols to interact with model membranes has been previously shown, where those that contain galloyl groups are more interactive than their non-galloylated precursors [13,17]. In particular, tellimagrandins I and II (Tel-I and Tel-II) and 1,2,3,4,6-penta-*O*-galloyl-d-glucose (PGG) (structures shown in Figure 1) have all been shown to have some form of interaction with lipid vesicles prepared from a phospholipid extract of *Escherichia coli* (*E. coli*) as a Gram-negative membrane model [10]. Therefore, we have selected these galloylated polyphenols alongside (–)-epigallocatechin gallate (EGCg) to give a series of polyphenols bearing different numbers of galloyl groups to study fundamental interactions with a simple Gram-negative bacterial membrane model using differential scanning calorimetry (DSC) as a precursor to selecting systems for further studies by neutron scattering to explore the structural changes in these model membranes during interactions [18].

While published studies have commonly employed *E. coli* as a model Gram-negative organism, bacterial membrane lipid composition is more diverse to the extent that a single species can display different membrane compositions [19]. Therefore, this study used fully hydrated 1,2-dipalmitoyl-sn-glycero-3-phosphorylethanolamine (DPPE)/ 1,2-dipalmitoyl-sn-glycero-3-phospho-(1’-rac-glycerol) (DPPG) (3:2 molar ratio) lipid vesicles as a simple Gram-negative bacterial membrane model. This two-component mixture of DPPE/DPPG (3:2) was selected based on a previously published meta-analysis of lipid headgroup composition of bacterial membranes [20]. Furthermore, the choice of lipid mixture in this work was selected to reflect the nature of the charges present in the bacterial lipid envelope and were primarily fabricated to exhibit non-ideal lipid mixing featuring lateral domains since these are often more representative of membranes found in nature [21].

## 2. Materials and Methods

### 2.1. Materials

DPPE and DPPG were obtained as powders from Avanti Polar Lipids (Alabaster, AL, USA). All lipids were used without further purification. HPLC-grade chloroform (Sigma Aldrich, Burlington, VT, USA) was added to the lipids and evaporated under N_2_-stream to form a dehydrated lipid cake. The lipid cake was rehydrated using HEPES (Sigma Aldrich, Burlington, USA)-based buffer with ultra-pure water (18 MΩ). The buffers were prepared at a concentration of 20 mM HEPES and adjusted to pH 7.2 using either 0.5 M NaOH or HCl, and also included CaCl_2_ (2 mM) and NaCl (100 mM). All lipid solutions were prepared to a concentration of 1 mg/mL and degassed for 10 min before use.

Tel-I and Tel-II were extracted from meadowsweet flowers (*Filipendula ulmaria*), isolated and purified by column chromatography on Sephadex LH-20 and preparative and semipreparative HPLC, and characterized by UHPLC-MS/MS and NMR spectroscopy according to previously published methods [22]. The purities of the Tel-I and Tel-II were 97.1% and 96.5%, respectively, as determined by UHPLC with UV detection at 280 nm. PGG was prepared from tannic acid, as described in Salminen and Lempa [23]. The purity of the PGG was 98.9%, as determined by UHPLC with UV detection at 280 nm [9]. (-)-EGCg (>95% purity) was purchased from Sigma Aldrich and was used as received. The structures of the polyphenols are shown in Figure 1.

### 2.2. Lipid Vesicle Preparation

We prepared small unilamellar vesicles (SUVs) by two methods to produce either ideally mixed or non-ideally mixed systems, with the difference between the two in DSC being characterized by the shape and number of DSC thermogram peak(s) observed. The extent of non-ideal mixing (i.e., de-mixing to exhibit phase-separated lateral domains of either lipid component) can be affected by the physical properties of the lipids, such as differing acyl chain lengths, acyl chain unsaturation, and headgroup charge repulsion, as well as through the vesicle preparation methods [24,25]. Ideally mixed lipid vesicles were prepared using 5 heating/cooling cycles (65 °C/5 °C respectively) with 1 min of vortexing in between each hot and cold phase to ensure homogeneous mixing. Non-ideally mixed lipid vesicles were prepared through sonication for 5 min at 20 kHz using a model 120 probe sonicator (Fischer Scientific, Waltham, MA, USA) at room temperature to produce a monodisperse vesicle solution. The SUVs were characterized by dynamic light scattering (DLS) measurements on a Zetasizer Nano (Malvern Panalytical, Malvern, UK), and the data were analyzed using Zetasizer software (version 8.02, Malvern Panalytical, UK) to confirm vesicle diameters below 100 nm (see the Supplementary Materials for data). The value of the vesicle refractive index was 1.47, the dispersant was HEPES buffer (R.I = 1.33, viscosity = 0.8872 mPa.s), and the temperature was 25 °C for all measurements. The lipid/polyphenol ratios refer to a % *w*/*w* concentration.

### 2.3. Differential Scanning Calorimetry

DSC measurements were carried out using a Nano DSC (TA instruments, New Castle, DE, USA) supported by NanoAnalyze software version 2.1. The lipid vesicles were prepared as above. Blank scans were recorded with HEPES buffer in both the reference and vesicle cells, and these were subtracted from the lipid thermograms. The scan rate was 1 °C/min, with a temperature range of 10–80 °C. Each vesicle scan was repeated in triplicate and found to be highly reproducible. The vesicles were prepared fresh from stock solutions (5 mg/mL) before each scan. The blank scans were subtracted from the vesicle scans, and the thermograms were normalized to the lipid concentration and volume. Offsets were applied to each scan shown in any given figure. In all cases, the thermogram displayed in the figure is the second of the set of repeats. When calculating the area under the peaks to derive the enthalpy and entropy values, integration was performed using a baseline of polynomial order 1 (see the Supplementary Materials for data).

## 3. Results and Discussion

### 3.1. DSC Thermograms of Individual Lipid Components

Differential scanning calorimetry (DSC) was used to investigate the effects of the polyphenolic compounds (-)-EGCg, Tel-I, Tel-II, and PGG on the model bacterial membrane vesicles. The model membranes were composed of DPPE/DPPG (3:2). The interactions were unambiguously shown through changes in the T_m_ of the lipids. In general, it was observed that as the concentration of polyphenol increased in the lipid vesicles, there was a temperature increase in the position of the transition peak associated with the DPPG and a temperature decrease in the peak position of the DPPE lipid.

This study clarifies the difference between two preparations for lipid vesicles both in terms of how the vesicles are physically prepared as well as their impact, which can be seen in the DSC scans. The lipids in these binary mixtures can be prepared such that they display either (i) ideal mixing with no, or very little, observable phase separation or (ii) non-ideal mixing, where the phase separation can be observed, but the micro-domains are unstable [26].

**Figure 2:**
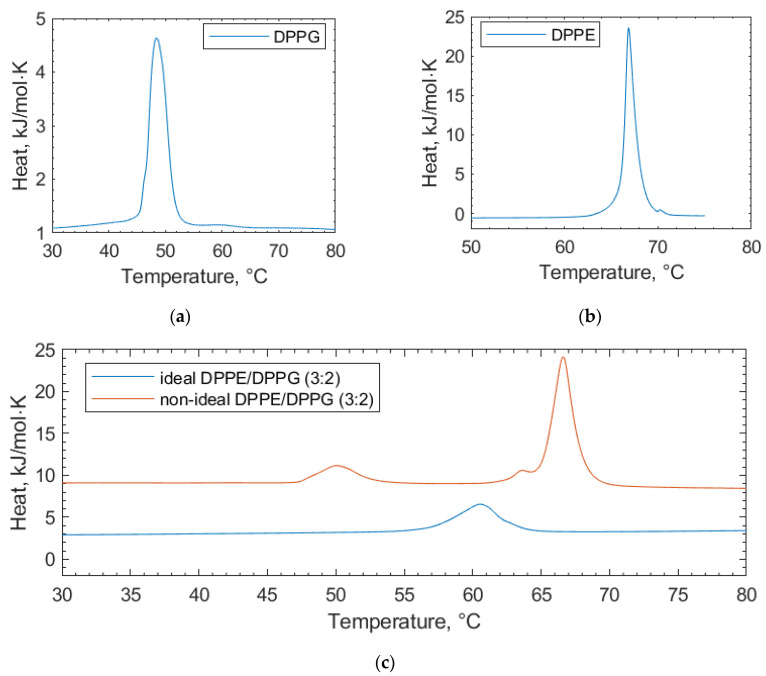
DSC thermograms of pure lipid components (**a**) DPPG and (**b**) DPPE used in the lipid mixtures for the bacterial membrane studies. (**c**) Ideally mixed and non-ideally mixed DPPE/DPPG (3:2) vesicles are shown, highlighting the differences in the transition temperatures depending on the preparation method. In all cases, the thermograms shown are the second scans recorded under heating.

In the scans of the non-ideally mixed DPPE/DPPG (3:2) (Figure 2), a shoulder is prevalent at the transition peak at 67 °C, which is indicative of non-ideal mixing and micro-domain formation [26]. Such a shoulder is not observed in the ideally mixed DPPE/DPPG (3:2) vesicles. In addition to vesicle preparation, non-ideal mixing can be affected by the physical properties of the lipids, such as differing chain lengths and headgroup charges [24]. Differences in the lipid headgroups, such as DPPG and DPPE compositions, can be enough to induce non-ideal mixing [25]. The transition temperatures of the individual lipid components we report are consistent with other values in the literature [27]. Increases in the lipid transition temperature are likely due to a stabilizing of the gel phase of the lipid through electrostatic interactions with the lipid headgroups [28]. Here, electrostatic interactions is an umbrella term used to encapsulate all manner of charge-based lipid–polyphenol interactions (van der Waals, dipole–dipole interactions, etc.) that cannot be individually resolved using DSC. Similarly, decreasing transition temperatures can be linked to the increasing size of some polyphenols, causing them to become more encumbered and less able to interact at the membrane surface [28]. Such size-based factors for interactions are termed “steric effects” in further discussion.

### 3.2. Interactions of (-)-EGCg with Ideally and Non-Ideally Mixed Lipid Vesicles

**Figure 3:**
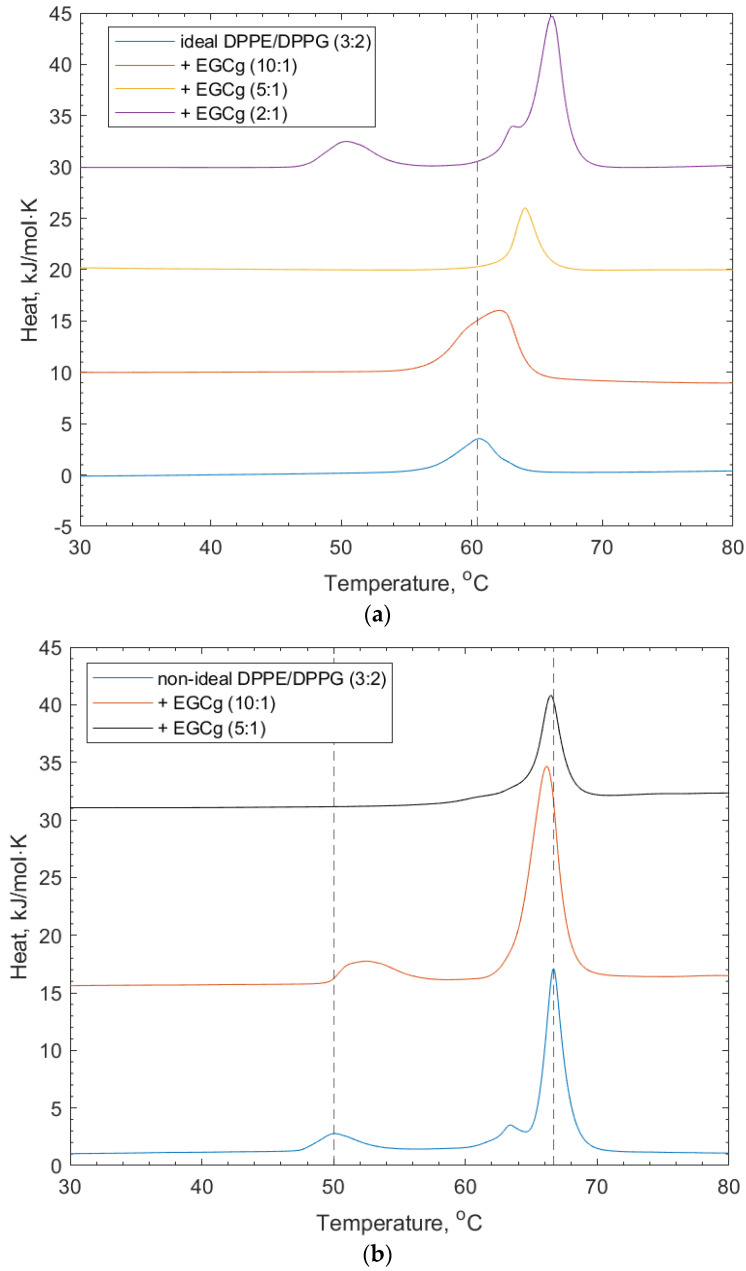
(**a**) DSC thermograms for ideally mixed DPPE/DPPG (3:2) + (-)-EGCg at increasing concentrations. The dotted line refers to the peak position of the lipid-only vesicles and illustrates the shift of the transition temperature and eventual (-)-EGCg-induced de-mixing of peaks. (**b**) DSC thermograms for non-ideally mixed DPPE/DPPG (3:2) with (-)-EGCg at increasing concentrations. The dotted lines indicate the reference positions of the DPPG peak (51.6 °C) and DPPE peaks (67 °C) in the lipid-only vesicles and illustrate the shifting of peaks as the concentration of (-)-EGCg increases.

For their interactions with (-)-EGCg, both ideally and non-ideally mixed lipid vesicles composed of DPPE/DPPG (3:2) were studied. The interaction of (-)-EGCg binding to the ideal lipid vesicles is shown in Figure 3 and Table 1. Lower concentrations of (-)-EGCg show a shifting of the lipid transition to higher temperatures, indicating that the presence of the polyphenol stabilized the gel phase of the lipid. A dotted line was added for reference at the position of the ideal DPPE/DPPG (3:2) vesicle to show the extent of the transition temperature shifting after the addition of (-)-EGCg. The highest (-)-EGCg concentration displays apparent de-mixing of the lipid peaks from the ideal peak, with the reappearance of the shoulder at the main DPPE-transition. The shoulder at the DPPE peak indicates the presence of non-uniformly mixed regions of the lipid membrane [26].

Figure 3 and Table 1 show that the non-ideally mixed lipid vesicles show signs of induced mixing with an increasing concentration of (-)-EGCg. The position of the DPPE–lipid-associated peak drops to a lower transition temperature (66.4 °C), which suggests that the mixture became less stable around the transition of the lipid phases; however, the DPPG-associated peak is not apparent at higher concentrations of (-)-EGCg. In addition, the shoulder on the DPPE feature becomes much broader and much lower in intensity. This suggests that, here, the (-)-EGCg induced disruption of the membrane microdomains that formed compared to the lipid vesicles without (-)-EGCg present. The increasing stability of the lipid gel phase may also have been influenced by the headgroup interactions with the polyphenol. As mentioned in the introduction, these vesicles were taken forward for the remaining lipid–polyphenol interaction studies, as they more accurately reflect the imperfect and asymmetric nature of membranes in a real cellular environment.

The ability of (-)-EGCg to exhibit such effects on lipid vesicles, which are reported to be notably more significant than other non-galloylated catechins, is ascribed to its galloyl moiety and number of hydroxyl groups enabling multiple hydrogen bonds to be formed with the polar headgroup of phospholipids [29].

### 3.3. Interactions of PGG, Tel-I, and Tel-II with Non-Ideally Mixed Lipid Vesicles

**Figure 4:**
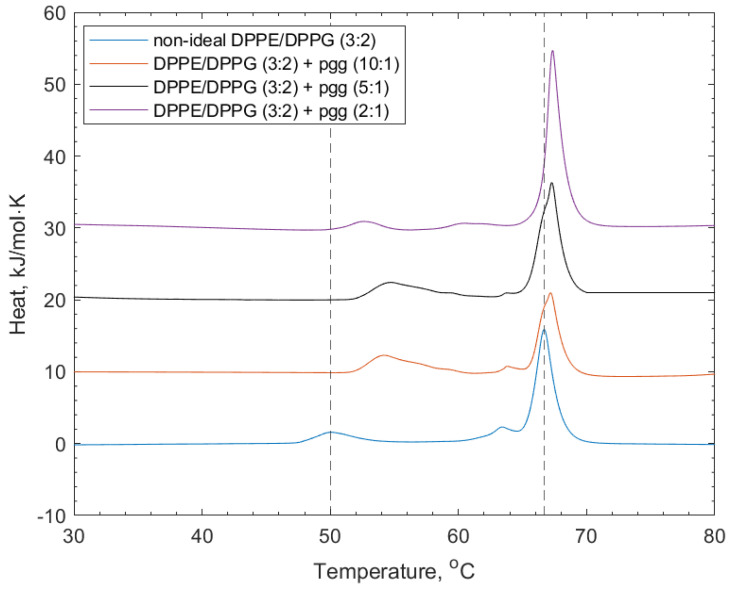
DSC thermograms showing non-ideal DPPE/DPPG (3:2) lipid vesicles, and their interactions with PGG at increasing concentrations. Dotted lines indicate the reference positions of the DPPG peak (51.6 °C) and DPPE peaks (67 °C) in the lipid-only vesicles and illustrate the shifting of peaks of the transition temperature upon the addition of PGG (see also Table 2).

Figure 4 shows the DSC thermograms of the interactions of PGG with the non-ideal mixed lipid vesicles. The addition of PGG to the lipid vesicles induced shifting of both the DPPG and DPPE lipid peaks to higher transition temperatures (52.9 and 67.4 °C respectively, see Table 2). The increase in the transition temperature is linked to the increased stability of the lipid phase before the transition temperature through the polyphenol–headgroup interactions. There was also a reduction in the intensity of the shoulder at the DPPE-associated feature, indicating induced mixing of the lipids.

**Figure 5:**
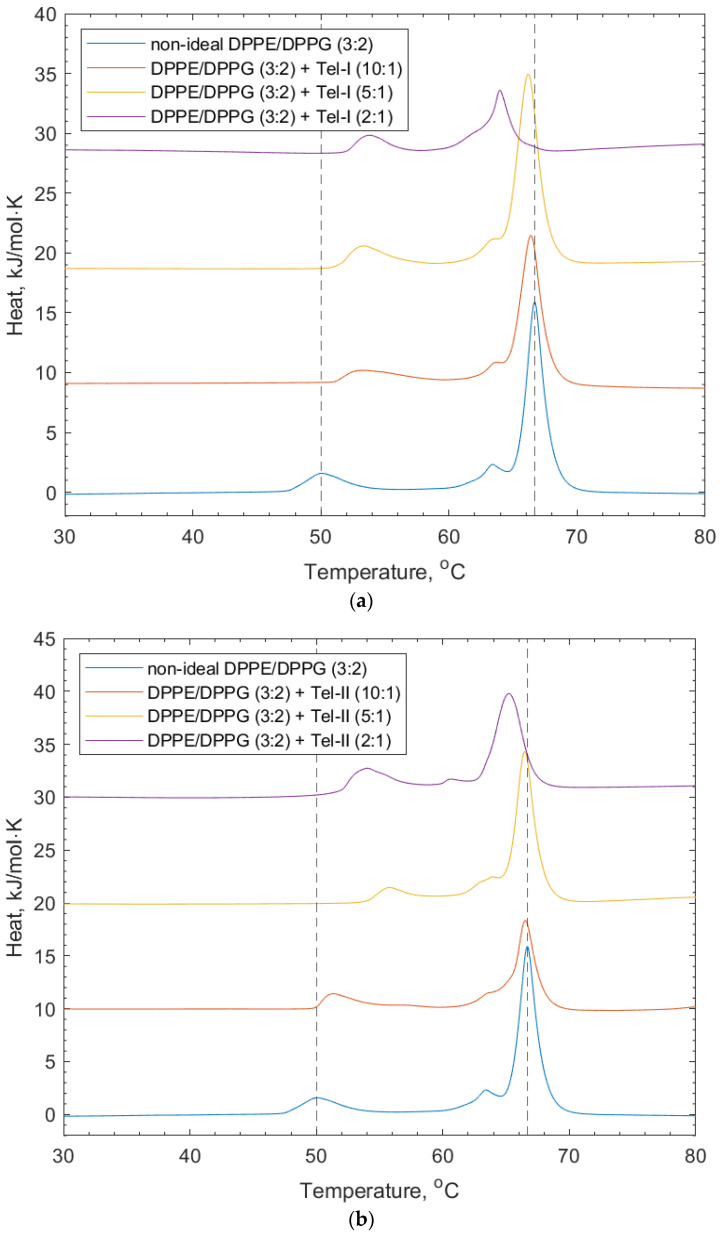
DSC thermograms showing non-ideal DPPE/DPPG (3:2) lipid vesicles, and their interaction with (**a**) Tel-I and (**b**) Tel-II. Dotted lines indicate the reference positions of the DPPG peak (51.6 °C) and DPPE peaks (67 °C) in the lipid-only vesicles and illustrate the shifting peaks of the transition temperature upon the addition of both Tel-I and Tel-II (see also Table 2).

Figure 5 shows the effects of Tel-I and Tel-II on the model bacterial membrane vesicles. The thermograms in Figure 5a show that the increasing concentration of Tel-I resulted in an increase in the transition temperature of the DPPG peak (+1.7 °C). This suggests that the Tel-II interacted with the DPPG in a way that stabilized the lipid phase, which is likely due to the intercalation of the galloyl groups into the membrane, similar to the PPG above. In contrast, a decrease was observed in the DPPE lipid peak (−1.7 °C). The shoulder associated with the DPPE lipid peak from the lipid-only scan also shifted to an area of a lower transition temperature. The decrease in the DPPE transition temperature may have arisen from the galloyl groups on the Tel-I molecule intercalating into the membrane, which can lead to localized changes in the lipid packing order [9,30]. Tel-I is more rigid than PGG, as it has one (*S*)-hexahydroxydiphenoyl (HHDP) group and two galloyl groups in its structure, so it is less able to adopt a conformation in the tail region, which allows for optimized lipid packing, resulting in a transition temperature decrease with respect to the DPPE.

In Figure 5b, the effects of Tel-II on the bacterial model membrane vesicles are shown. Similarly for Tel-I, there was an increase in the transition temperature of the DPPG peak (+1.7 °C) and a decrease for the DPPE (−1.6 °C). Tel-II also has one (*S*)-HHDP group in its structure and three freely rotating galloyl groups. As can be seen in Figure 1, the absence of free galloyl at the O1 position of the central glucose in Tel-I made it less hydrophobic than Tel-II. As before, the increase in the position of the DPPG peak is related to the charge-based headgroup interactions with Tel-II.

Table 3 provides a summary of the lipid peak shifts and some key physical properties of each polyphenol. The logP values of the polyphenols given in Table 3 can be used as a guide for their propensity to interact with the lipids, although the hydrophobicity of polyphenols does not fully determine their ability to penetrate the lipid membranes. It is understood from NMR studies that these interactions take place predominantly with the headgroups of the lipids [9]. However, it should be noted that lipid–polyphenol interactions can take place in various places across and through the lipid bilayer [30]. Polyphenolic compounds of this kind have been shown to interact electrostatically at the membrane surface with lipid headgroups [31,32]. While the logP values of all four of the polyphenols discussed here could not be found in the same literature source, the (-)-EGCg logP values used were determined by HPLC-MS methods [33,34].

Tel-I and Tel-II had the lowest logP values and both caused a decrease in the DPPE lipid transition temperature, which is attributed to their intercalating into the vesicle membrane and disrupting the optimal lipid packing. The increase in the transition temperature for the DPPG lipid component can be explained by the charge-based headgroup interactions with the hydroxyl group on the galloyl moieties. PGG shows transition temperature increases in both the DPPE and DPPG lipids, which indicates that interactions with both lipids is related to the stabilizing headgroup interactions.

The transition temperature shift caused by the PGG to the DPPG lipid was larger than that for DPPE, suggesting that there is a preference for interaction with DPPG over DPPE. The immiscibility of lipid components can facilitate preferential interaction with one lipid component over another [25]. There are signs of the lipid–polyphenol interactions preferring the DPPG lipid component for charge-based interactions, which is justified through the ease of access to hydrogen bond donors and acceptors at the DPPG headgroup. The interaction with PGG did not result in a reduction of the transition temperature for either lipid component. This indicates that there was no intercalation of PGG into the membrane, which can be explained by considering its bulky and non-planar structure. 

(-)-EGCg resulted in a decrease in the DPPE lipid transition temperature, which is again likely due to intercalation into the membrane. The DPPG transition temperature increases with the initial addition of (-)-EGCg, but at high concentrations, a DPPG peak was not observed. This is explained as an interruption of the membrane micro-domains by the addition of (-)-EGCg. The lower magnitude of the temperature shift on the non-ideally mixed lipid vesicles through the (-)-EGCg addition can be attributed to having fewer galloyl groups than the other polyphenols studied.

The results of the effect of polyphenols on lipid T_m_ demonstrate that having more galloyl groups within the polyphenol structure increases the ability of the polyphenol to interact, with molecular flexibility and logP dictating the extent of the polyphenol intercalation into the membrane [13]. In the case of PGG, having more galloyl groups did not result in a significantly larger interaction, as there came a point where the bulk of the PGG molecule prevented further membrane interaction. These trends follow those observed in isothermal titration calorimetry studies of polyphenol–lipid and polyphenol–protein interactions, with bovine serum albumin as the protein [10].

## 4. Conclusions

The studies presented above elucidate the interactions among a series of polyphenolic compounds, including (-)-EGCg, Tel-I, Tel-II, and PGG and a model bacterial membrane composed of DPPE/DPPG (3:2) using differential scanning calorimetry (DSC). When compared to the pure lipid mixture, the interactions with vesicles led to observable shifts in the DSC transition temperature peaks. Notably, we chose to focus on non-ideally mixed lipid vesicles for our interaction studies, thus simulating the inherent imperfections in lipid mixing found in natural membranes and facilitating the formation of equilibrated microdomains.

The nature of these interactions was analyzed in relation to various factors, including the number of galloyl groups available for membrane interaction, the logP values, and the structural flexibility of the polyphenols. Our findings suggest a preference of the polyphenols for the DPPG lipid component within the mixture. This preference is attributed to the capacity of DPPG to engage in hydrogen bonding and other electrostatic dipole–dipole interactions with the four polyphenols under investigation. This research sheds light on the underlying mechanisms governing polyphenol–membrane interactions and their dependence on specific structural and electrostatic characteristics, contributing to a deeper understanding of these complex processes.

## Figures and Tables

**Table 1:** Mean values, standard deviation, and standard error values for the transition temperatures of ideally mixed DPPE/DPPG (3:2) + (-)-EGCg relating to the thermograms for the ideal and non-ideal DPPE/DPPG (3:2) shown in Figure 3.

Lipid Vesicle System	Peak 1/°C	Std. Dev. (n = 3)	Peak 2/°C	Std. Dev. (n = 3)
Ideal DPPE/DPPG (3:2)	60.51	0.17	-	-
+ (-)-EGCg (10:1) *	62.10	0.07	-	-
+ (-)-EGCg (5:1)	63.99	0.15	-	-
+ (-)-EGCg (2:1)	50.46	0.23	66.12	0.06
Non-ideal DPPE/DPPG (3:2)	51.60	0.60	67.00	0.10
+ (-)-EGCg (10:1)	52.00	0.50	66.20	0.10
+ (-)-EGCg (5:1)	-	-	66.40	0.10

* Ratio of lipid vesicle to polyphenol.

**Table 2:** Mean values for the transition temperatures of non-ideally mixed DPPE/DPPG (3:2) + PGG, +Tel-I, or +Tel-II, relating to the thermograms shown in Figure 4 and Figure 5. Data are given with their associated standard deviation values.

Lipid Vesicle System	Peak 1/°C	Std. Dev. (n = 3)	Peak 2/°C	Std. Dev. (n = 3)
Non-ideal DPPE/DPPG (3:2)	51.60	0.60	67.00	0.10
+ PGG (10:1)	57.00	1.20	65.70	0.10
+ PGG (5:1)	55.90	0.80	66.20	0.90
+ PGG (2:1)	52.90	0.20	67.40	1.10
+ Tel-I (10:1)	50.60	0.10	66.50	0.10
+ Tel-I (5:1)	52.00	0.80	66.10	0.10
+ Tel-I (2:1)	53.25	0.73	65.26	1.36
+ Tel-II (10:1)	53.00	0.80	66.40	0.10
+ Tel-II (5:1)	54.60	1.30	66.50	0.10
+ Tel-II (2:1)	53.30	0.40	65.40	0.20

**Table 3:** Summary table showing the direction of the DPPG and DPPE peak shifts for ideal and non-ideally mixed DPPE/DPPG (3:2) vesicles and associated peak shifts upon interaction with each polyphenol.

Polyphenol	DPPG Peak Shift	Mean DPPG Peak Temp Shift ^a^/°C	DPPE Peak Shift	Mean DPPE Peak Temp Shift ^a^/°C	Number of Free Galloyl Groups	Nominal Mass	LogP
(-)-EGCg	-	-	Decrease	−0.6	1	442 Da	1.89 ^b^
Tel-I	Increase	+1.7	Decrease	−1.7	2	786 Da	−0.44 ^c^
Tel-II	Increase	+1.7	Decrease	−1.6	3	938 Da	0.86 ^c^
PGG	Increase	+1.3	Increase	+0.4	5	940 Da	1.49 ^c^

^a^ Mean peak shifts are given for the lipid/polyphenol = 2:1 vesicle system. ^b^ Reference [34]. ^c^ Reference [22].

## Data Availability

The data presented in this study are available within the article and supplementary materials.

## Supplementary Materials

The following supporting information can be downloaded at: https://www.mdpi.com/article/10.3390/membranes14020047/s1. The following supporting data are provided. Figure S1: Typical number-weighted particle size distribution for SUVs produced from 3:2 DPPE/DPPG lipid mixes; Table S1: Thermodynamic parameters from reported DSC data.
