# Supplementary material for: Interactions of Galloylated Polyphenols with a Simple Gram-Negative Bacterial Membrane Lipid Model
Source: Membranes (Basel). 2024 Feb 8;14(2):47. doi: 10.3390/membranes14020047 (PMC10890094; doi:10.3390/membranes14020047)
Supplement: Supplementary file 1 [file membranes-14-00047-s001.zip › membranes-2831284-supplementary.pdf]

Supplementary Materials for Membranes

Interactions of galloylated polyphenols with a simple Gram-negative bacterial membrane lipid model

Ryan T. Coones [1], Maarit Karonen [2], Rebecca J. Green [1,*] and Richard Frazier [1]

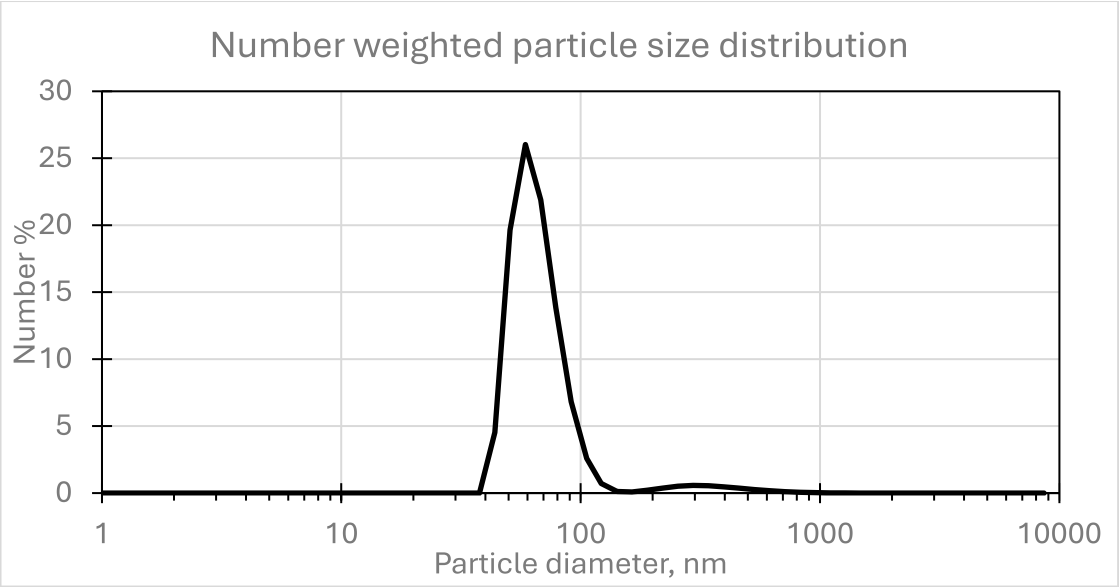

Figure S1. Typical number weighted particle size distribution for SUVs produced from 3:2 DPPE/DPPG lipid mixes.

Table S1. Thermodynamic parameters from reported DSC data

Sample	Peak 1			Peak 2		
	ΔH, kJ/mol	ΔS, kJ/mol.K	Integration limits, °C	ΔH, kJ/mol	ΔS, kJ/mol.K	Integration limits, °C
DPPE/DPPG (3:2) non-ideal mixing	7.4342	0.023	46-57	33.52	0.10	60 - 72
DPPE/DPPG (3:2) Ideal mixing	-	-	-	13.65	0.04	52 - 68
DPPE/DPPG (3:2) + EGCg (10:1) ideal mix	-	-	-	33.67	0.10	52-68
DPPE/DPPG (3:2) + EGCg (5:1) ideal mix	-	-	-	14.69	0.04	56-70
DPPE/DPPG (3:2) + EGCg (2:1) ideal mix	10.5367	0.0326	45-57	43.07	0.13	58-72
DPPE/DPPG + egcg (10:1)	8.2665	0.0254	47-59	50.33	0.15	60-72
DPPE/DPPG + egcg (5:1)	-	-	-	22.69	0.07	55-72
DPPE/DPPG + pgg (10:1)	10.9875	0.0336	50-63	23.15	0.07	62-72
DPPE/DPPG + pgg (5:1)	9.8094	0.0299	50-61	30.12	0.09	63-70
DPPE/DPPG + pgg (2:1)						
DPPE/DPPG + pgg (2:1)	3.4398	0.0106	47-57	42.21	0.12	58-73
DPPE/DPPG + Tel-II (10:1)	3.09	0.0095	49-57	24.41	0.07	57-70
DPPE/DPPG + Tel-II (5:1)	2.6241	0.008	52-59	30.79	0.09	60-72
DPPE/DPPG + Tel-II (2:1)	7.0131	0.0214	49-59	21.32	0.06	62-70
DPPE/DPPG + Tel-I (10:1)	4.5921	0.0141	50-60	28.78	0.08	60-71
DPPE/DPPG + Tel-I (5:1)	5.7588	0.0176	49-59	37.58	0.11	60-71
DPPE/DPPG + Tel-I (2:1)	3.8901	0.0119	50-58	12.40	0.04	58-69
